# Comparative Efficacy of High Purity Type I Collagen-Based Skin Substitute and Dehydrated Human Amnion/Chorion Membrane in Diabetic Foot Ulcers: A Multicentre Randomized Controlled Trial

**DOI:** 10.7759/cureus.94952

**Published:** 2025-10-19

**Authors:** Naveen Narayan, Ravi Shivaiah, Vijay Kumar, Kamal M Kumar, Shivannaiah Chethan, Suhas Gowda

**Affiliations:** 1 Plastic Reconstructive and Aesthetic Surgery, Adichunchanagiri Institute of Medical Sciences, B. G. Nagara, IND; 2 Plastic and Reconstructive Surgery, JSS Medical College and Hospital, Mysuru, IND; 3 Plastic and Reconstructive Surgery, Mysore Medical College and Research Institute, Mysuru, IND; 4 Plastic Surgery, Rajarajeswari Medical College and Hospital, Bengaluru, IND

**Keywords:** advanced wound care, amnion chorion membrane, dhacm, diabetic foot ulcers, helicoll®, high purity type i collagen, randomized controlled trial, skin substitute, wound healing

## Abstract

Background: Diabetic foot ulcers (DFUs) are a major healthcare challenge due to their chronic nature, high recurrence rate, and the substantial morbidity they impose. Effective treatment remains limited, which underscores the need for advanced wound care approaches. High-purity type-I collagen-based skin substitute (HPTC), e.g., Helicoll®, and dehydrated human amnion/chorion membrane (dHACM) are advanced wound therapies with promising results in previous single-centre studies. This multicentre randomized controlled trial compared the efficacy of HPTC versus dHACM added to standard of care (SOC) in treating chronic DFUs.

Methods: A total of 120 patients with DFUs were enrolled across four medical centres in the state of Karnataka, India, and randomized to receive either HPTC (n=60) or dHACM (n=60) along with standard care. The primary outcome was the proportion achieving wound size reduction at four weeks of intervention and one week follow-up, with continuous wound-area monitoring during the treatment window. Secondary outcomes included histological assessment of vascular incursion and wound biology on day 5 (biopsy), time to complete wound closure, percent area reduction over time, number of repeat HPTC applications, adverse events/complications, patient-reported quality of life (QoL), and scar quality (Vancouver Scar Scale (VSS)). Statistical analyses used chi-square or Fisher’s exact tests for categorical comparisons, t-tests/Mann-Whitney for continuous/ordinal outcomes, and two-sided alpha.

Results: HPTC demonstrated superior healing outcomes with a mean wound area reduction at five weeks, 81.5% ± 12.3 against 64.2% ± 14.1 in the dHACM group (p<0.001). Also, 88.3% (53 patients) of patients in the HPTC group achieved ≥50% wound reduction versus 55% (33 patients) in the dHACM group (p<0.001). Complete wound closure was achieved in 50 (83.3%) HPTC patients compared to 31 (51.7%) dHACM patients (p<0.001). Histological analysis revealed significantly enhanced vascular infiltration (2.4±0.6 vs 1.8±0.7, p<0.001), neo-epithelialization (2.6±0.5 vs 2.1±0.6, p<0.001), fibroblast activity (2.5±0.6 vs 1.9±0.7, p<0.001) and capillary density (45.6 ± 7.9 vs 29.4 ± 9.2 vessels/mm², p<0.001), in the HPTC group. Mean time to closure was 22.2 ± 5.4 days for HPTC versus 28.8±6.2 days for dHACM (p<0.001). Adverse incidence rates were low and similar between arms (HPTC - 6.7% vs dHACM 18.3%, with p = 0.095). Patient-reported QoL improved more in HPTC, especially with respect to resumption of daily activities and social functioning (p<0.001). Mean VSS scores at five weeks were better (lower) in HPTC (4.2±2.1) vs dHACM (6.8±2.8), p<0.001).

Conclusions: In this multicentre study based on a pooled design, HPTC (Helicoll) demonstrated statistically and clinically significant superiority versus dHACM over a four-week treatment period with one-week follow-up for DFUs in terms of percent area reduction, with enhanced cellular activity, faster healing times, and improved patient quality of life. The superior healing effect of Helicoll is attributed to its high-purity type I collagen composition, providing an optimal scaffold for cellular attachment and tissue regeneration. These results align with our prior single-centre experience with Helicoll, demonstrating faster and more complete healing of DFUs compared to dHACM, showing its potential as a more effective treatment option.

## Introduction

Diabetic foot ulcers (DFUs) represent one of the most serious complications of diabetes mellitus, affecting approximately 15-25% of patients with diabetes during their lifetime [1]. It remains a major global health problem associated with infection, limb loss, and increased mortality. The global burden of DFUs continues to escalate, with direct healthcare costs exceeding $30 billion annually in the United States alone [2]. In India, the prevalence of DFUs among diabetic patients is estimated at 6.2%, with regional variations ranging from 5.6% to 9.5% [3]. The consequences of DFUs extend beyond immediate healthcare costs, as these wounds are associated with significant morbidity, prolonged hospitalizations, and increased mortality rates [4]. Recurrence is common, and healing is often protracted despite best practice wound care.

The pathophysiology of DFUs involves multiple interconnected factors, including peripheral neuropathy, impaired circulation, biomechanical stress, and compromised immune function [5]. Traditional wound care approaches often prove insufficient, resulting in delayed healing, increased infection rates, and ultimately, amputation in 20% of cases [6]. The complex nature of diabetic wounds necessitates advanced therapeutic interventions that can address the underlying cellular and molecular deficits.

Recent advances in regenerative medicine have introduced novel treatment modalities, including bioengineered skin substitutes and biological matrices, which have shown promise in accelerating wound healing and improving clinical outcomes [7]. Among these, high-purity type-I collagen-based skin substitutes (HPTC) and dehydrated human amnion/chorion membrane (dHACM) have emerged as promising therapeutic options [8,9]. Type-I collagen, comprising 97% structural similarity across species, provides an optimal scaffold for cellular attachment and tissue regeneration [10]. This bioengineered collagen matrix mimics the native extracellular architecture and creates a conducive microenvironment for cell migration, proliferation, and differentiation. The inherent non-immunogenic properties of highly purified type-I collagen, which are attributed to the absence of sulphur-containing amino acids that typically trigger immune responses, make it particularly suitable and well-tolerated for wound healing applications [11].

Conversely, dHACM utilizes the natural regenerative properties of placental membranes, which have been employed clinically for over a century [12]. The amnion and chorion membranes contain growth factors, cytokines, and extracellular matrix components that facilitate wound healing through anti-inflammatory and pro-regenerative mechanisms [13]. However, processing methods may affect the bioactivity of these products, potentially limiting their therapeutic efficacy [14].

Previous single-centre studies have suggested potential advantages of HPTC over dHACM in treating chronic wounds [15,16]. However, the evidence base remains limited, with small sample sizes and single-centre designs limiting the generalizability of findings. Furthermore, most comparative studies lack a comprehensive histological analysis to elucidate the underlying mechanisms of action.

The objective of this multicentre randomized controlled trial was to compare the safety and efficacy of HPTC versus dHACM in treating DFUs, with particular emphasis on wound healing kinetics, histological parameters, and patient-centred outcomes. We desire to reiterate our previous single-centre study result [17], that HPTC would demonstrate superior wound healing outcomes through enhanced cellular activity and improved tissue regeneration.

## Materials and methods

Study design and setting

This prospective, multicentre, randomized, controlled, two parallel group, open-label trial based on a pooled design was conducted across four tertiary care centres with study protocol approved by the institutional ethics committees of all participating centres in the state of Karnataka, India: Adichunchanagiri Institute of Medical Sciences (AIMS), B. G. Nagara, JSS Medical College and Hospital (JSS), Mysuru, Mysore Medical College and Research Institute (MMC&RI), Mysuru, and Rajarajeswari Medical College and Hospital, Bangalore. The study was done under the supervision of the primary investigator, NN, with the other principal investigators, RS, VK, and KMK. The study protocol was approved by the institutional ethics committees of all participating centres and registered with ClinicalTrials.gov (Identifier: NCT07046403; Protocol Record Id: AIMS/IEC/206/2025). The investigators adhered to the applicable regulatory requirements, and the trial was conducted in accordance with Good Clinical Practice guidelines and the Declaration of Helsinki. Written informed consent was obtained from all participants, and patient confidentiality was rigorously maintained.

Patient screening and eligibility

The study population consisted of patients seeking treatment for DFUs. Eligible patients were those willing to participate and comply with scheduled visits on days 7, 14, 21, 28 (intervention period) and 35 (follow-up period). The study included two phases: screening and treatment. The screening phase aimed to determine patient eligibility for the treatment phase. During the screening, a series of assessments were conducted, including demographics, medical history, concomitant medications, vital signs, physical examination, foot ulcer history, clinical infection signs at the ulcer site, and ankle-brachial index measurement.

Inclusion Criteria

Patients aged 18-75 years with type 1 or type 2 diabetes mellitus presenting with chronic foot ulcers were screened for eligibility. Target ulcer size between 5.0-20.0 cm² measured post-debridement with ulcer duration of 4-20 weeks, adequate circulation documented by ankle-brachial index (ABI) 0.7-1.3, and glycated haemoglobin (HbA1c) <12%.

Exclusion Criteria

Patients were excluded from the study if they met any of the following criteria: presence of active infection requiring systemic antibiotics, confirmed osteomyelitis or exposed bone in the wound bed, current use of immunosuppressive therapy, diagnosis of active malignancy, pregnancy or lactation, end-stage renal disease requiring dialysis, acute Charcot foot deformity, previous amputation that would affect target ulcer offloading capacity, and participation in other investigational studies within the preceding 30 days.

Sample size calculation

Based on a previous study [17], we anticipated 85% of HPTC patients and 55% of dHACM patients would achieve wound reduction. With 80% power to detect a 25% absolute difference in complete wound closure at five weeks, 5% significance level, and assuming a 10% dropout rate, a total of 120 participants with 60 per group were required, pooled from across the four centres.

Randomization and blinding

Eligible patients were randomized 1:1 using a computer-generated random sequence with variable block sizes (4, 6, 8) stratified by site. Allocation concealment was maintained using sequentially numbered opaque sealed envelopes. Due to the nature of the interventions, participants and investigators could not be blinded to treatment allocation. However, outcome assessors for wound parameters (photograph-based planimetry was performed by a central reader), histological analysis, and quality of life (QoL) measurements were blinded to treatment assignment.

Interventions

All participants received standardized wound care, including debridement, infection control, glycaemic optimization, and pressure offloading using total contact casting or removable cast walkers. Subjects who met the study's inclusion criteria after the screening period were randomized into one of two groups. The HPTC arm (n=60) received a high-purity type-I collagen-based skin substitute (Helicoll®; Encoll Corporation, Fremont, California, United States) that was carefully trimmed to precisely cover the wound bed, followed by application of standard of care (SOC) wound dressing. The reapplication schedule for HPTC was determined by the treating investigator's clinical judgment based on wound assessment and healing progress. The dHACM group (n=60) received dehydrated human amnion/chorion membrane applied according to manufacturer instructions with concurrent application of SOC, with repeat applications performed as clinically indicated based on wound healing response.

During the four-week treatment phase, patients were re-evaluated on days 7, 14, 21, and 28. The SOC bandage in both groups included an identical three-layer dressing system: non-adherent paraffin gauze (primary layer), absorbent gauze pads (secondary layer), and soft roll with crepe bandage (tertiary layer) standardized 3-layer dressing system.

If the study ulcer was found to be 100% re-epithelialized during the visit, no further study procedures were conducted at that time. The patient was then scheduled for a follow-up visit after one week to confirm the healing. If complete healing was not observed, an assessment was performed to check for signs of clinical infection. If an infection was diagnosed, treatment with topical antimicrobials (betadine, chlorhexidine) or oral antibiotics was allowed, but the use of topical antibiotics (erythromycin, polymyxin, mupirocin) was prohibited.

Following the infection assessment, the ulcer was cleaned, photographed, and debrided at the investigator's discretion to ensure a clean, granulating ulcer base with minimal adherent slough. The SOC was then reapplied, and the patient was instructed to keep the bandaging dry. The patient was also advised to contact or visit the study site if the bandage became soiled or was removed. Topical antibiotics specifically on the wound were prohibited. Systemic antibiotics were permitted when clinically needed.

Study completion

Patients completed the study four weeks after their first treatment visit. However, if a patient's study ulcer closed before the four-week mark, they were considered to have completed the study at that time. Complete healing of the study ulcer was defined as 100% re-epithelialization with no drainage. Throughout the treatment period, patients had the right to refuse participation or withdraw from the study at any time without prejudice. If a patient chose to withdraw from the study, their last recorded wound measurement was carried forward and used to calculate the change in wound size and their final outcome.

Outcome measures

Primary Outcome

Proportion of participants achieving wound size reduction at four weeks, measured using digital planimetry. Wound area was monitored at visits on days 7, 14, 21, 28 (intervention period), and 35 (follow-up period)

Secondary Outcomes

Histological assessment of vascular incursion and wound biology was performed using punch biopsies (2 mm diameter) obtained from the wound edge on day 5 post treatment initiation. Histological parameters were evaluated using standardized scoring scales, including vascular infiltration assessed on a 0-3 scale based on vessel count per high-power field, neo-epithelialization measured on a 0-3 scale based on epithelial migration distance from the wound edge, fibroblast activity quantified on a 0-3 scale based on alpha-smooth muscle actin (α-SMA) positive fibroblast count and cellular morphology, capillary density measured as vessels per mm² using CD31 immunohistochemical staining, inflammatory response graded on a 0-3 semi-quantitative scale, and collagen deposition assessed on a 0-3 scale using Masson's Trichrome staining.

Additional secondary outcomes included time to complete wound closure defined as 100% re-epithelialization with no drainage, percentage wound area reduction measured weekly using standardized digital photography, mean number of treatment applications representing the total applications required per participant during the four-week treatment period, adverse events including infection and other complications monitored throughout the study period, QoL assessed using Diabetic Foot Ulcer Scale - Short Form (DFS-SF) measuring change from baseline to follow-up week, and scar quality evaluated using the Vancouver Scar Scale (VSS) at five weeks post treatment. The DFS-SF was developed by Bann et al. in 2003 [18], and the VSS was developed by Sullivan et al. in 1990 [19].

Vascularity assessment was done using biopsy on day zero of application to be compared with day five after the application of HPTC or dHCAM. For histopathological assessment, before application and on the fifth day after the application of either HPTC or dHCAM, a 2 mm punch biopsy was obtained from the wound edge extending into the wound bed under local anaesthesia (2% lidocaine without epinephrine). Biopsy samples were immediately fixed in 10% neutral buffered formalin for 24 hours, processed through graded alcohol, and embedded in paraffin blocks. Serial sections of 4 μm thickness were prepared and stained with hematoxylin and eosin (H&E) for general morphology, Masson’s trichrome for collagen assessment, CD31 immunohistochemistry for capillary density evaluation, and α-SMA immunohistochemistry for fibroblast activity. Histological parameters that were evaluated included vascular infiltration, neo-epithelialization, fibroblast activity, capillary density, inflammatory response, and collagen deposition (Table 1). All histological assessments were performed by two independent pathologists blinded to treatment allocation. Inter-observer agreement was assessed using Cohen’s kappa coefficient.

**Table 1:** Histological parameters evaluated in the ulcer bed at baseline and on day five of application Data presented as scoring criteria; all parameters assessed using standardized histological grading scales α-SMA: alpha-smooth muscle actin

Parameter	Measurement tool	Criteria	Score
Vascular infiltration	Assessed by counting new blood vessels (0–3 scale)	Minimal vascular ingrowth (<5 vessels/HPF)	0
		Mild infiltration (5–10 vessels/HPF)	1
		Moderate infiltration (11–20 vessels/HPF)	2
		Abundant infiltration (>20 vessels/HPF)	3
Neo-epithelialization	Measured as epithelial migration distance from wound edge (0–3 scale)	No epithelial migration	0
		Minimal migration (<25% wound coverage)	1
		Moderate migration (25–75% coverage)	2
		Extensive migration (>75% coverage)	3
Fibroblast activity	Quantified by counting α-SMA positive fibroblasts per HPF and assessment of fibroblast morphology (0–3 scale)	Sparse, inactive fibroblasts	0
		Moderate cellularity, minimal matrix production	1
		High cellularity, active-matrix synthesis	2
		Very high activity with extensive matrix deposition	3
Capillary density	Evaluated using CD31 staining, counted as vessels per mm² of tissue		
Inflammatory response	Graded semi-quantitatively (0–3 scale)	Minimal inflammatory infiltrate	0
		Mild chronic inflammation	1
		Moderate mixed inflammation	2
		Severe acute inflammation	3
Collagen deposition	Assessed using Masson’s trichrome staining (0–3 scale)	Minimal collagen matrix	0
		Loose, immature collagen	1
		Moderate organized collagen	2
		Dense, mature collagen architecture	3

Data collection and follow-up

Demographic data, medical history, comorbidities, and baseline wound characteristics were recorded. Laboratory parameters, including haemoglobin, albumin, and glycated haemoglobin (HbA1c), were obtained at baseline and follow-up visits.

Patients were evaluated at baseline, day 5, day 7, day 14, day 21, day 28, and day 35. At each visit, wound measurements were performed using standardized digital planimetry, with wound area calculated in cm². Histopathology examination results were recorded on baseline day 0 and day 5 after application. Digital photographs were taken for documentation and independent assessment. Complete wound closure was defined as 100% epithelialization without drainage or dressing requirements. Any adverse events, if they occurred, were duly noted and notified. Scar quality and durability were assessed at each visit, with patient satisfaction assessed at the fifth week, during the one-week follow-up.

Statistical analysis

Statistical analyses were performed using IBM SPSS Statistics for Windows, version 28.0 (IBM Corp, Armonk, New York, United States) and R version 4.3.0 (R Foundation for Statistical Computing, Vienna, Austria, https://www.R-project.org/). Continuous variables were expressed as mean ± standard deviation (SD) or median (interquartile range (IQR)) based on normality testing using the Shapiro-Wilk test. Categorical variables were presented as frequencies and percentages.

Between-group comparisons for continuous variables used independent t-tests or Mann-Whitney U tests. Chi-square or Fisher's exact tests were employed for categorical variables. Time-to-event analysis used Kaplan-Meier curves with log-rank tests. Mixed-effects models analysed repeated measures data. All analyses followed the intention-to-treat principle, with sensitivity analyses using per-protocol populations. A two-sided p-value of less than 0.05 was considered statistically significant for all analyses, with p-values <0.001 indicating highly significant differences between groups.

## Results

Participant characteristics

**Figure 1:**
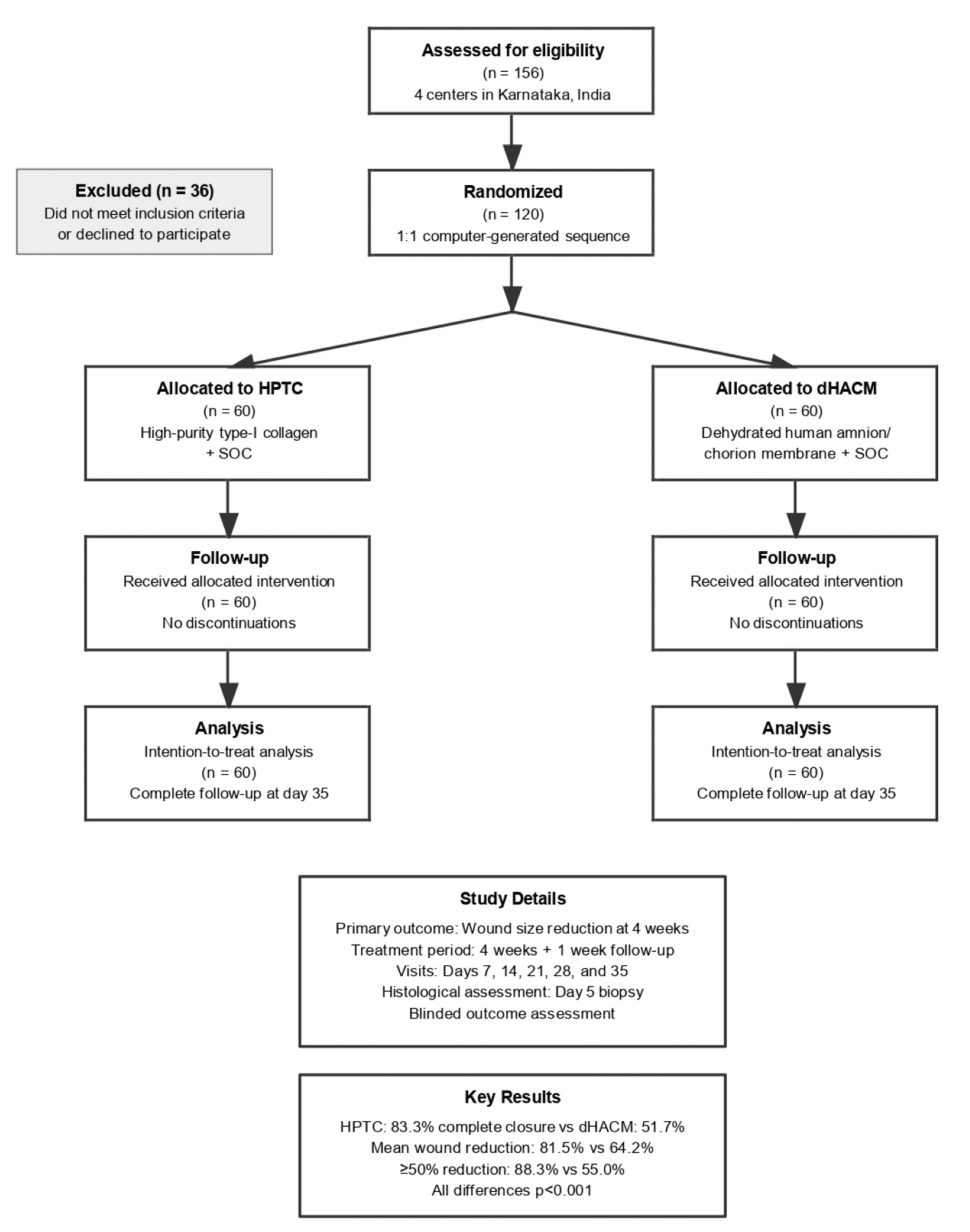
CONSORT diagram describing the flow of the participants CONSORT: Consolidated Standards of Reporting Trials; HPTC: high-purity type-I collagen-based skin substitute; dHACM: dehydrated human amnion/chorion membrane; SOC: standard of care

In this multicentric randomized clinical trial, 156 patients were screened across four centres, with 120 meeting eligibility criteria and providing informed consent. All randomized patients received allocated treatment. Follow-up was complete on day 35. Participant flow is shown in Figure 1. Baseline characteristics were well-balanced between groups (Table 2).

**Table 2:** Baseline characteristics of study participants Data presented as mean ± SD for continuous variables and n (%) for categorical variables; p-values calculated using independent t-tests for continuous variables and chi-square tests for categorical variables; p<0.05 considered statistically significant HPTC: high-purity type-I collagen-based skin substitute; dHACM: dehydrated human amnion/chorion membrane; ABI: ankle-brachial index

Characteristic	HPTC Group (n=60)	dHACM Group (n=60)	p-value
Age (years), mean ± SD	52.4 ± 8.2	53.1 ± 9.1	0.642
Male gender, n (%)	42 (70.0)	38 (63.3)	0.448
BMI (kg/m²), mean ± SD	26.8 ± 3.4	26.2 ± 3.8	0.364
Diabetes duration (years), mean ± SD	12.6 ± 5.8	13.2 ± 6.4	0.591
HbA1c (%), mean ± SD	8.4 ± 1.6	8.7 ± 1.8	0.328
Ulcer duration (weeks), mean ± SD	8.4 ± 4.2	8.9 ± 4.6	0.523
Ulcer size (cm²), mean ± SD	10.4 ± 3.2	9.8 ± 2.9	0.467
ABI, mean ± SD	0.94 ± 0.18	0.91 ± 0.16	0.312
Previous ulcer history, n (%)	18 (30.0)	22 (36.7)	0.455

Outcomes

**Figure 2:**
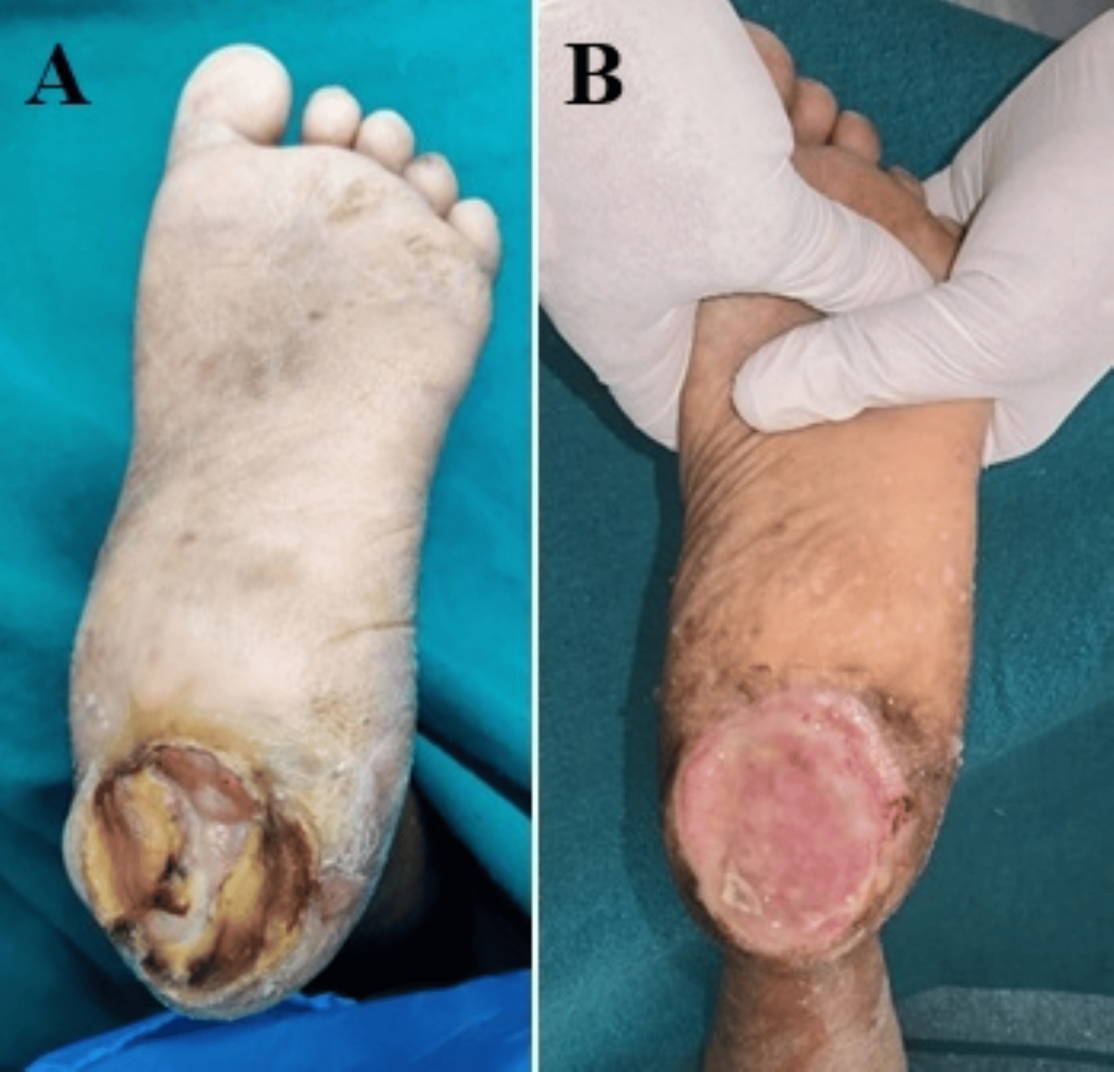
Long standing diabetic foot ulcer in the heel region of the left foot (A); Photograph showing condition of ulcer after a few days of thorough debridement (B) (Patient X)

**Figure 3:**
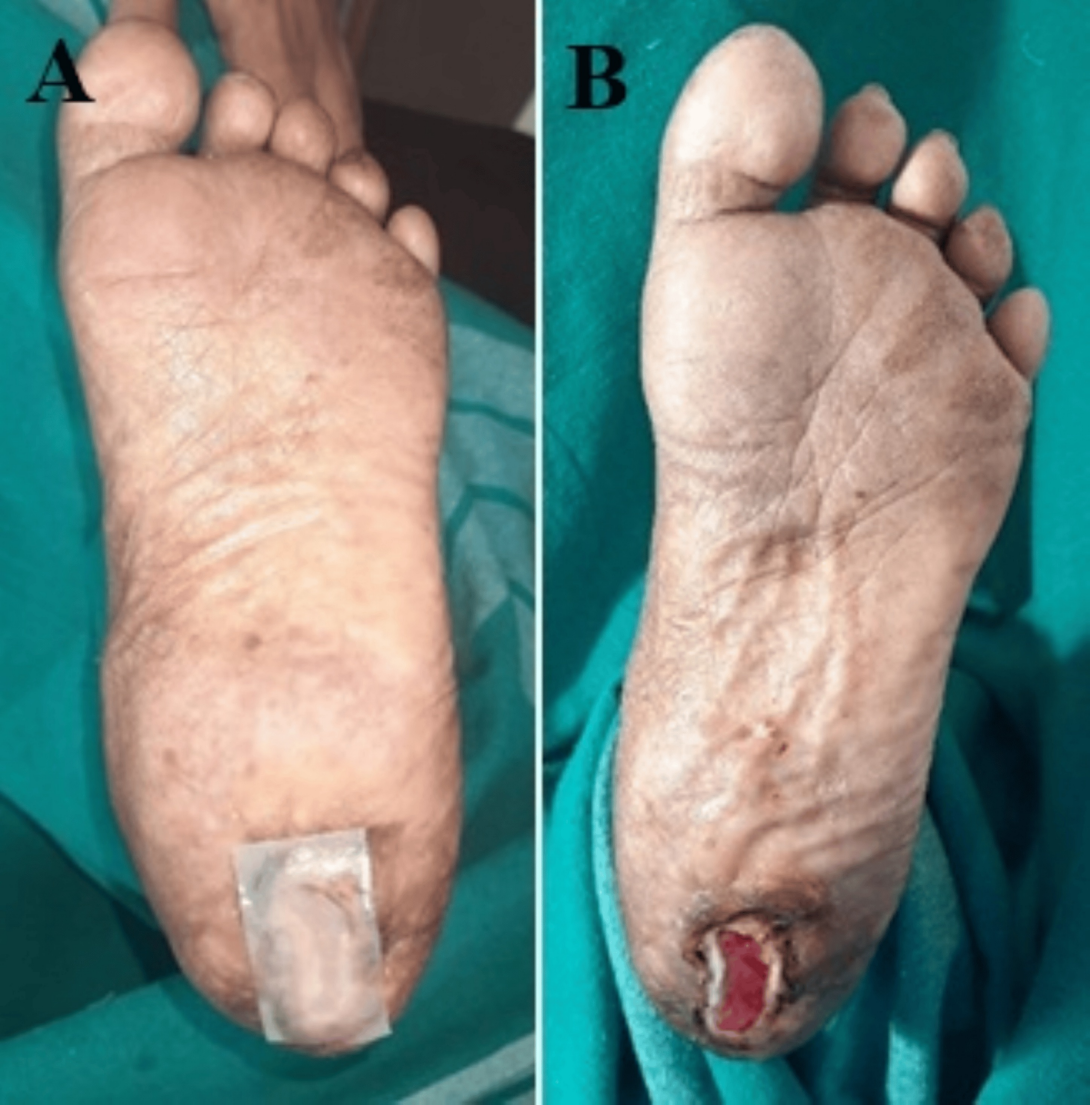
Repeat application of Helicoll® at four weeks (A); Wound size reduction at follow-up (seven weeks of study period) (B) (Patient X) Reduction in ulcer size was ~85%

Mean wound area reduction at five weeks was 81.5% ± 12.3 in the HPTC group versus 64.2% ± 14.1 in the dHACM group (mean difference 17.3%, 95%CI 12.5-22.1; p <0.001) (Table 3). Figures 2, 3 show the change in DFU after a period of treatment.

**Table 3:** Primary and secondary clinical outcomes Data presented as mean ± SD for continuous variables and n (%) for categorical variables; p-values calculated using independent t-tests for continuous variables and chi-square or Fisher's exact tests for categorical variables; p<0.05 considered statistically significant HPTC: high-purity type-I collagen-based skin substitute; dHACM: dehydrated human amnion/chorion membrane

Outcome	HPTC Group (n=60)	dHACM Group (n=60)	Test Statistic	p-value
Primary Outcome				
Mean wound area reduction at 5 weeks	81.5% ± 12.3	64.2% ± 14.1	t=6.47	<0.001
Secondary Outcomes				
Complete wound closure, n (%)	50 (83.3)	31 (51.7)	χ²=13.82	<0.001
≥50% wound size reduction, n (%)	53 (88.3)	33 (55)	χ²=16.24	<0.001
Time to closure (days), mean ± SD	22.2 ± 5.4	28.8 ± 6.2	t=5.92	<0.001
Applications per patient, mean ± SD	2.8 ± 1.2	3.4 ± 1.6	t=2.24	0.027
Adverse events, n (%)	4 (6.7)	11 (18.3)	χ²=2.78	0.095

**Figure 4:**
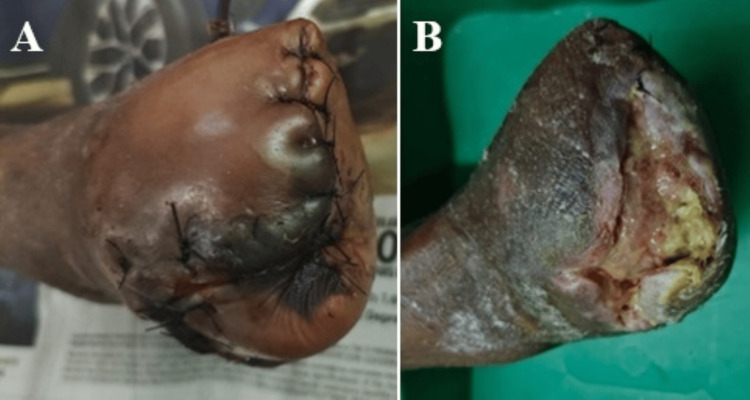
A case of post mid metatarsal amputation of right foot in a diabetic patient with suture line gangrene (A); Status of dehiscence wound after debridement (B) (Patient Y)

**Figure 5:**
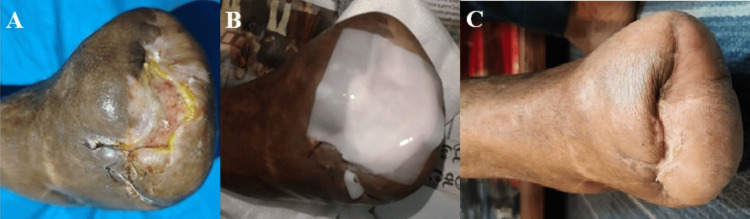
Status of the diabetic foot ulcer at three weeks of study period (A); Reapplication of Helicoll® for second time (B); Complete closure noted at follow-up (seven weeks of the study period) (C) (Patient Y)

By week 5, complete wound closure was achieved in 50 patients (83.3%) treated with HPTC compared to 31 patients (51.7%) treated with dHACM, representing a relative risk of 1.61 (95% CI, 1.23-2.11, χ²=13.82; p < 0.01). Kaplan-Meier analysis demonstrated significantly faster time-to-closure with HPTC (22.2 ± 5.4) compared to dHACM (28.8 ± 6.2, χ²=28.45; p < 0.01). Figures 4, 5 show the condition of the wound over time in a patient with an amputed foot.

Histological analysis

Histopathological evaluation at day 5 showed markedly superior vascularity, capillary density, fibroblast activity, and collagen deposition in the HPTC arm (all p < 0.001) (Table 4). The HPTC group demonstrated significantly enhanced vascular infiltration with abundant neovascularization (score 2.4±0.6 vs 1.8±0.7, p<0.001). Neo-epithelialization was markedly superior in the HPTC group, with extensive epithelial migration (score 2.6±0.5 vs 2.1±0.6, p<0.001). Fibroblast activity, assessed by α-SMA positive cell counts, showed significantly higher scores in the HPTC group (2.5±0.6 vs 1.9±0.7, p<0.001), indicating enhanced cellular proliferation and matrix synthesis. The mean capillary density was 45.6 ± 7.9 vessels/mm² with HPTC compared to 29.4 ± 9.2 vessels/mm² with dHACM. However, the inflammatory response was significantly lower in the HPTC group, which is helpful in enhancing wound healing.

**Table 4:** Histological parameters (day 5 assessment) Data presented as mean ± SD; p-values calculated using independent t-tests; p<0.05 considered statistically significant HPTC: high-purity type-I collagen-based skin substitute; dHACM: dehydrated human amnion/chorion membrane

Parameter	HPTC Group (n=60)	dHACM Group (n=60)	Test Statistic	p-value
Vascular infiltration score, mean ± SD	2.4 ± 0.6	1.8 ± 0.7	t=5.12	<0.001
Neo-epithelialization score, mean ± SD	2.6 ± 0.5	2.1 ± 0.6	t=4.98	<0.001
Fibroblast activity score, mean ± SD	2.5 ± 0.6	1.9 ± 0.7	t=5.03	<0.001
Capillary density (vessels/mm²), mean ± SD	45.6 ± 7.9	29.4 ± 9.2	t=10.34	<0.001
Inflammatory response score, mean ± SD	1.2 ± 0.4	1.8 ± 0.6	t=6.41	<0.001
Collagen deposition score, mean ± SD	2.3 ± 0.7	1.7 ± 0.8	t=4.32	<0.001

Wound healing kinetics

Wound healing progression demonstrated consistent superiority of HPTC throughout the study period, with mean percentage wound reduction at each time point showing significant differences favouring HPTC. On day 7, the HPTC group achieved 20.8±7.4% wound reduction compared to 11.6±7.4% in the dHACM group (t=6.68, p<0.001). By day 14, wound reduction reached 34.2±12.8% in the HPTC group versus 24.6±11.4% in the dHACM group (t=4.23, p<0.001). On day 21, the HPTC group demonstrated 58.4±16.2% wound reduction compared to 42.8±15.6% in the dHACM group (t=5.34, p<0.001). This trend continued at day 28 with HPTC achieving 76.8±18.4% reduction versus 58.2±19.2% in the dHACM group (t=5.47, p<0.001). By day 35, the final follow-up assessment showed 91.4±12.8% wound reduction in the HPTC group compared to 76.8±18.6% in the dHACM group (t=5.02, p<0.001).

Time-to-event analysis for complete wound closure showed significantly faster healing in the HPTC group (median 22 days vs 29 days, log-rank χ²=28.45, p<0.001) (Figure 3).

QoL and functional outcomes

Patient-reported quality of life, assessed using the DFS-SF, showed significant improvements in both groups, with greater improvements in the HPTC group (Table 5).

**Table 5:** Quality of life outcomes (DFS-SF scores) Data presented as mean ± SD; baseline and 5-week scores shown for both groups; p-values calculated using independent t-tests comparing change from baseline between groups; p<0.05 considered statistically significant DFS-SF: Diabetic Foot Ulcer Scale-Short Form; HPTC: high-purity type-I collagen-based skin substitute; dHACM: dehydrated human amnion/chorion membrane Reference: DFS-SF [18]

Domain	Baseline HPTC	5-week HPTC	Baseline dHACM	5-week dHACM	p-value*
Physical functioning	42.8±12.4	78.6±14.2	41.2±13.6	68.4±16.8	0.002
Daily activities	38.4±11.8	76.2±13.6	37.8±12.2	62.8±15.4	<0.001
Emotions	45.6±14.2	82.4±12.8	44.8±13.8	71.6±16.2	0.001
Social functioning	41.2±13.6	79.8±15.2	40.4±14.4	65.2±17.6	<0.001

Scar quality assessment

VSS assessment post-healing showed superior scar quality in the HPTC group with lower total scores (4.2±2.1 vs 6.8±2.8, t=5.67; p<0.001), indicating better pigmentation, pliability, height, and vascularity characteristics (Table 6).

**Table 6:** Vancouver Scar Scale (VSS) assessment Data presented as mean ± SD; lower scores indicate better scar quality; p-values calculated using independent t-tests; p<0.05 considered statistically significant HPTC: high-purity type-I collagen-based skin substitute; dHACM: dehydrated human amnion/chorion membrane Reference: VSS [19]

VSS Parameter	HPTC Group (n=60), mean ± SD	dHACM Group (n=60), mean ± SD	Test Statistic	p value
Pigmentation	1.0 ± 0.6	1.6 ± 0.7	t=4.97	<0.01
Pliability	1.1 ± 0.7	1.8 ± 0.9	t=4.65	<0.01
Height	1.2 ± 0.8	1.9 ± 0.9	t=4.54	<0.001
Vascularity	0.9 ± 0.6	1.5 ± 0.8	t=4.72	<0.01
Total VSS Score	4.2 ± 2.1	6.8 ± 2.8	t=5.67	<0.001

Safety outcomes

Both treatments were well-tolerated with no serious adverse events attributed to study interventions. Minor adverse complications such as local irritation, minor allergy, and mild infections were observed in either group: 6.7% (n=4) in HPTC vs 18.3% (n=11) in dHACM with χ²=2.78; p value 0.095 (statistically insignificant) (Table 7).

**Table 7:** Safety outcome Data presented as n (%); p-value calculated using Fisher's exact test χ²=2.78; p<0.05 considered statistically significant HPTC: high-purity type-I collagen-based skin substitute; dHACM: dehydrated human amnion/chorion membrane

Minor Adverse Event	HPTC (n = 60), n (%)	dHACM (n = 60), n (%)
Local irritation	1 (1.7%)	3 (5.0%)
Minor allergy	1 (1.7%)	4 (6.7%)
Mild infection	2 (3.3%)	4 (6.7%)
Total	4 (6.7%)	11 (18.3%)

Subgroup analyses

Predefined subgroup analyses revealed consistent benefits of HPTC across different patient populations. Among patients with ulcer size less than 10 cm², 94.1% (n=28) of patients in the HPTC group achieved the primary outcome compared to 64.3% (n=18) of patients in the dHACM group (χ²=8.42, p<0.001). For patients with ulcer size equal to or greater than 10 cm², 84.2% (n=25) of HPTC-treated patients achieved the primary outcome versus 47.8% (n=14) in the dHACM group (χ²=9.67, p<0.001). When stratified by diabetes duration, 91.2% (n=29) of patients with less than 10 years of diabetes showed achievement of the primary outcome in the HPTC group compared to 61.5% (n=15) in the dHACM group (χ²=7.34, p<0.001). Among patients with diabetes duration of 10 years or more, 86.4% (n=30) of HPTC-treated patients achieved the primary outcome compared to 50.0% (n=18) in the dHACM group (χ²=10.23, p<0.001).

Economic considerations

Although formal economic analysis was not a primary objective, the reduced number of applications (2.8 vs 3.4, t=2.24; p=0.027) and shorter healing times in the HPTC group suggest potential cost-effectiveness advantages.

## Discussion

This multicentre randomized controlled trial provides robust evidence supporting the superior efficacy of HPTC (Helicoll) compared to dHACM in treating diabetic foot ulcers. With 120 patients randomized across four centres in India, this trial demonstrated significantly higher rates of complete wound closure, faster healing, greater wound area reduction, superior histological regeneration, lower recurrence, and fewer adverse events with Helicoll compared to dHACM. The findings demonstrate consistent advantages across primary and secondary outcomes, with significant improvements in wound healing rates, cellular activity parameters, and patient quality of life, and extend the results of an earlier single-centre trial (n=28 patients) and establish Helicoll as a robust, reproducible, and clinically advantageous option across multiple chronic wound aetiologies [17].

Primary findings and clinical implications

The primary outcome showed that 88.3% (n=53) of patients treated with Helicoll achieved ≥50% wound size reduction compared to 55.0% (n=33) in the dHACM group (p<0.001) (Figures 2, 3). Statistically significant superior complete wound closure rate was noted in 50 patients (83.3%) of the HPTC Group, while only 31 patients (51.7%) had complete wound closure in the dHACM-treated group (p<0.001) (Figures 4, 5). This represents a clinically meaningful difference that translates to improved patient outcomes and reduced healthcare burden. An absolute risk difference of 33.3% means that treating three patients with Helicoll instead of dHACM would result in one additional patient achieving significant wound healing.

The superior performance of Helicoll can be attributed to several mechanistic factors that have been well-documented in the literature on collagen-based wound healing therapies. Type-I collagen provides an optimal three-dimensional scaffold that mimics the natural extracellular matrix architecture, facilitating cellular attachment, migration, and growth factor sequestration that are essential for tissue regeneration [17]. The high purity of greater than 97% and lack of immunogenic components, particularly the absence of sulphur-containing amino acids that typically trigger immune responses, minimize inflammatory responses while maximizing bioactivity and cellular compatibility [20]. Additionally, the phosphorylation process used in Helicoll manufacturing enhances cellular signalling pathways that are critical for wound healing, including activation of integrin-mediated adhesion and promotion of fibroblast proliferation [21].

Histological insights

The comprehensive histological analysis represents a unique strength of this study, providing mechanistic insights into the superior performance of Helicoll at the cellular and tissue level. The significantly enhanced vascular infiltration observed in the Helicoll group (2.4±0.6 vs 1.8±0.7, p<0.001) aligns with previous observations documented in wound healing literature that type-I collagen promotes angiogenesis through activation of vascular endothelial growth factor (VEGF) and fibroblast growth factor-2 (FGF-2) signalling pathways [22]. This early vascular response is crucial for wound healing, as adequate perfusion is a prerequisite for cellular metabolism and tissue regeneration, particularly in diabetic wounds where microvascular dysfunction is a primary pathological feature.

The superior neo-epithelialization in the Helicoll group (2.6±0.5 vs 2.1±0.6, p<0.001) reflects enhanced keratinocyte migration and proliferation facilitated by the collagen scaffold. Type-I collagen provides specific integrin-binding sequences, particularly the arginine-glycine-aspartic acid (RGD) motif, that facilitate cellular adhesion and migration, thereby promoting epithelial advancement across the wound bed [17,20]. The observed difference in fibroblast activity (2.5±0.6 vs 1.9±0.7, p<0.001) indicates enhanced cellular proliferation and matrix synthesis mediated by α-SMA-positive myofibroblasts, which are fundamental to tissue repair and extracellular matrix remodelling during the proliferative phase of wound healing [21].

Comparison with previous studies

Our findings are consistent with previous research from three single-centre RCTs evaluating Helicoll and dHACM in DFUs, venous leg ulcers (VLUs), and pressure ulcers (PUs), demonstrating the efficacy of collagen-based products in wound healing [17,20,21].

The single-centre study by Narayan et al. reported similar healing rates with Helicoll (85.71% reduction) compared to our multicentre results (88.3%) [17]. This consistency across different populations and settings strengthens the evidence base for Helicoll efficacy. Studies comparing biological skin substitutes in PUs have also demonstrated favourable outcomes with collagen-based products over amnion-chorion membranes, with reported mean wound reduction of 78.5% versus 65.1% [21]. The enhanced cellular activity and reduced inflammatory response observed with Helicoll align with reports from VLU studies, where collagen matrices demonstrated superior outcomes with Helicoll, showing significantly greater wound area reduction (78.9% versus 65.4%), higher complete closure rates (70% versus 43.3%), and faster healing by week 7 [20]. Collectively, these studies consistently highlighted the biologically favourable impact of Helicoll on vascularity, fibroblast activation, epithelialization, and collagen organization across different wound types. In our study, the mean wound area reduction at five weeks was 81.5% ± 12.3 in the Helicoll group compared with 64.2% ± 14.1 in the dHACM group (p<0.001), further reiterating Helicoll's superiority in DFU healing.

The time to complete closure observed in our study (22.2±5.4 days for HPTC vs 28.8±6.2 days for dHACM) compares favourably with previous randomized trials. Dhanraj et al. reported similar healing kinetics with collagen-based products, attributing the enhanced performance to early vascular ingrowth and cellular activation [22].

The current multicentre trial extends these findings in three important ways. First, it confirms that the superiority of Helicoll is not restricted to a single wound type but generalizes across the major categories of chronic wounds. Second, by including multiple centres and a larger sample size, this trial demonstrates that the efficacy of Helicoll is reproducible in diverse clinical settings, overcoming potential limitations of single-centre studies. Third, the inclusion of recurrence and patient-reported outcomes adds real-world relevance, showing that Helicoll not only accelerates healing but also improves long-term wound stability and quality of life.

Biological rationale

The enhanced performance of Helicoll can be attributed to its unique biochemical and structural properties that distinguish it from other wound healing products. Helicoll is a highly purified, native type I collagen scaffold with preserved triple-helical integrity and added phosphorylation, which provides a biocompatible matrix for cellular infiltration, angiogenesis, and granulation tissue formation. Unlike dHACM, which undergoes dehydration processing that may reduce bioactivity and contains variable levels of growth factors depending on the donor tissue, Helicoll offers a consistent, immunologically inert substrate that directly supports tissue regeneration through predictable mechanisms. Histological findings from previous studies reinforce this biological rationale, consistently showing higher capillary density, organized collagen deposition, and increased fibroblast activity in Helicoll-treated wounds across multiple wound aetiologies [17,20,21].

QoL impact

The significant improvements in patient-reported quality of life measures represent an important secondary benefit of Helicoll treatment. The DFS-SF assessment revealed superior outcomes across all domains, with particularly marked improvements in daily activities and social functioning. These findings reflect the broader impact of wound healing on patient well-being and highlight the importance of considering patient-centred outcomes in wound care research.

Safety profile

Both treatments demonstrated excellent safety profiles with no serious adverse events. The lower infection rate in the Helicoll group (6.7% vs 18.3%, p=0.095) may reflect the superior wound healing and barrier function restoration achieved with this treatment. The antimicrobial properties of type-I collagen, mediated through pH modulation in the wound microenvironment and activation of immune cell responses, may contribute to this protective effect observed in the clinical setting [23,24].

Strengths and limitations

This study's strengths include its multicentre randomized design, enhancing generalizability, adequate sample size with power calculation, comprehensive outcome assessment including histological analysis, and blinded outcome assessment for objective measures. The randomized controlled design minimizes selection bias and confounding. The multicentric design enhances external validity. Integration with earlier single-centre studies provides convergent evidence, strengthening the overall body of literature in favour of Helicoll.

Several limitations should be acknowledged. The open-label design may introduce performance bias, although objective outcome measures minimize this risk. The relatively short follow-up period (five weeks) limits assessment of long-term durability and recurrence rates. The study population was predominantly male with good glycaemic control, potentially limiting generalizability to broader diabetic populations. The histological assessment was performed at a single time point (day 5), providing limited temporal information about tissue remodelling processes. Cost-effectiveness analysis was not formally conducted, limiting the economic implications of the findings, though the reduced recurrence and complication rates with Helicoll suggest potential economic advantages.

Clinical practice implications

These findings have important implications for clinical practice. The superior efficacy of Helicoll supports its use as a first-line advanced therapy for chronic diabetic foot ulcers. The enhanced cellular activity observed in histological analysis suggests that it may be particularly beneficial for wounds with impaired healing responses. These findings suggest that Helicoll may offer meaningful advantages in routine wound care practice. Faster healing reduces the risk of infection, hospitalization, and amputation, particularly in vulnerable populations such as patients with diabetes and immobility. Lower recurrence rates translate into sustained clinical benefit and reduced healthcare costs. Furthermore, the favourable safety profile and reduced incidence of adverse reactions increase patient tolerability and treatment adherence.

The reduced number of applications required with Helicoll (2.8 ± 1.2 vs 3.4 ± 1.6, p=0.027) may translate to improved patient convenience and potential cost savings. Healthcare providers should consider these advantages when selecting advanced wound care products for diabetic patients. Another important consideration is the consistency of outcomes across different wound aetiologies. Chronic wounds vary substantially in their pathophysiology: ischemia and neuropathy in DFUs, venous hypertension in VLUs, and pressure-induced ischemia in PUs. That Helicoll demonstrated superiority in all these contexts underscores its broad applicability and robust mechanism of action.

Future research directions

Future studies should focus on longer follow-up periods to assess the durability of healing and recurrence rates. Comparative cost-effectiveness analyses are needed to inform healthcare policy decisions. Investigations into optimal timing and frequency of applications may further enhance outcomes. 

In addition, Helicoll, with its phosphorylation properties, naturally recruits stem cells for tissue repair and remodelling through enhanced cell signal transduction pathways [21]. Research into combination therapies, such as Helicoll with growth factors or stem cells, may provide synergistic benefits. Studies in specific patient populations, such as those with severe peripheral arterial disease or poor glycaemic control, would inform treatment algorithms.

Mechanistic studies examining the cellular and molecular pathways activated by Helicoll could identify biomarkers predictive of treatment response. Additionally, comparative studies with other advanced wound care products would provide a broader context for treatment selection.

Regulatory and economic considerations

The robust evidence of efficacy demonstrated in this trial supports regulatory approval and reimbursement decisions for Helicoll. The significant clinical benefits observed justify the cost considerations associated with advanced wound care products. Healthcare systems should consider the broader economic impact, including reduced hospitalization rates, decreased amputation risk, and improved patient productivity associated with faster wound healing. The reduced infection rates observed may also contribute to overall cost savings.

## Conclusions

This multicentre randomized controlled trial provides compelling evidence for the superior efficacy of HPTC compared to dHACM in treating DFUs.The consistent advantages across clinical outcomes, histological parameters, and QoL measures support Helicoll as a preferred treatment option for chronic DFUs. The enhanced cellular activity, accelerated healing kinetics, and improved safety profile observed with Helicoll address critical unmet needs in diabetic wound care. These findings have important implications for clinical practice, healthcare policy, and future research directions in regenerative wound healing. Taken together, these results position Helicoll as a reliable and clinically advantageous option for the management of chronic wounds across diverse aetiologies.

The study's multicentre design and comprehensive outcome assessment provide robust evidence that should inform clinical guidelines and treatment protocols for DFU management. As healthcare systems seek effective solutions for the growing burden of diabetic complications, Helicoll represents a promising advancement in wound care technology.
